# Chronic Urticaria Treatment with Omalizumab—Verification of NLR, PLR, SIRI and SII as Biomarkers and Predictors of Treatment Efficacy

**DOI:** 10.3390/jcm12072639

**Published:** 2023-04-01

**Authors:** Bartłomiej Tarkowski, Julia Ławniczak, Katarzyna Tomaszewska, Marcin Kurowski, Anna Zalewska-Janowska

**Affiliations:** 1 Psychodermatology Department, Medical University of Lodz, 92-213 Lodz, Poland; katarzyna.tomaszewska@umed.lodz.pl (K.T.); anna.zalewska-janowska@umed.lodz.pl (A.Z.-J.); 2 Faculty of Medicine, Medical University of Lodz, 92-213 Lodz, Poland; julia.lawniczak@stud.umed.lodz.pl; 3 Department of Immunology and Allergy, Medical University of Lodz, 92-213 Lodz, Poland; marcin.kurowski@umed.lodz.pl

**Keywords:** chronic spontaneous urticaria, omalizumab, predictors

## Abstract

Biomarkers that are able to predict the response to omalizumab (OMA) in chronic spontaneous urticaria (CSU) are highly valued. The aim of our study was to evaluate the UAS7 (urticaria activity score assessed for 7 days), DLQI (dermatology life quality index), SII (systemic immune-inflammation index), SIRI (systemic inflammation response index), PLR (platelet/lymphocyte ratio) and NLR (neutrophil/lymphocyte ratio) in a group of 46 CSU a patients treated for 24 weeks with OMA (300 mg every 4 weeks). There were no statistically significant differences observed at the start nor at the end of the treatment between the two groups (responders vs. non-responders) and SII, SIRI, PLR and NLR. However, a statistically significant correlation was observed between severity of urticaria expressed in UAS7 scores and the quality of life (evaluated by DLQI). Furthermore, at week 24, both groups demonstrated significant improvement in quality of life. Our single center study did not confirm the usefulness of SII, SIRI, NLR or PLR as predictors of the response to OMA in CSU. However, it is of importance that even patients who did not respond to the treatment presented a significant improvement in quality of life. Additionally, we also observed that the efficacy of treatment was unchanged amongst patients who underwent a second series of treatment in cases of relapse.

## 1. Introduction

Chronic urticaria (CU) is a condition characterized by appearance of wheals, angioedema or both, daily or intermittently, for more than 6 weeks. The symptoms can occur spontaneously, without definite triggers, as in chronic spontaneous urticaria (CSU). CU affects approximately 1% of the population and can have a great impact on the patient’s quality of life. It can disturb work, daily activities, sleep and social interactions [1,2].

Second generation antihistamines are the basis of therapy and, if necessary, can be administered in fourfold dosages. If the latter are not sufficient, omalizumab (OMA) can be added. OMA, a monoclonal anti-IgE antibody, was found to be safe and effective in the management of symptoms and the improvement of patient functioning [1].

The pathogenesis of urticaria is complex. The activation of mast cells and release of their mediators are known to play a crucial role, but other abnormalities are also observed. Autoimmunity, inflammation, complement system and coagulation cascades, as well as the links between them, are discussed [3]. Therefore, researchers investigate different hematological and inflammatory parameters, as well as their relationships to CSU severity and treatment response. Literature data point to interleukin-6 (IL-6) mean platelet volume (MPV), C-reactive protein (CRP), the platelet/lymphocyte ratio (PLR), and the neutrophil/lymphocyte ratio (NLR) as potential biomarkers of inflammation in many chronic diseases [4,5,6,7]. Some authors have found them useful in urticaria assessment. The need for further research is however also widely emphasized.

Literature data report the effects of OMA on blood parameters, such as a decrease in CRP, PLR and NLR, as well as differences between responders and non-responders [5,8,9]. The identification of helpful predictive biomarkers in urticaria is one of the aims set by international organizations. Importantly, biomarkers should be repeatable and applicable in clinical practice. Recent research has indicated some new markers to predict the response to OMA in CSU [9]. SIRI (the systemic inflammation response index) and SII (the systemic immune-inflammation index) are indexes based on the levels of neutrophils, lymphocytes and, accordingly, monocytes or platelets. They were also explored as markers and predictors in other diseases and conditions, such as different kinds of cancers or cardiovascular events [9,10]. Coşansu et al. demonstrated a significant decrease in the above parameters after OMA treatment, as well as differences in their levels between responders and non-responders [9]. SIRI and SII were suggested as novel cost-effective biomarkers in CSU and predictors for response to biologic therapy. The authors, however, concluded that further studies are needed to examine these relationships.

## 2. Materials and Methods

The aim of our study was to investigate whether levels of SII, SIRI, PLR, NLR or scores of UAS7 or DLQI can be useful in predicting the patients response to OMA therapy.

Our study included data collected from 56 CSU patients treated with OMA for 24 weeks (300 mg every 4 weeks) in the National Health Fund drug program since 2020. Every 4 weeks, a follow-up visit was performed with the assessment of urticaria severity (UAS7) and quality of life (DLQI). At the 1st, 4th and 6th visit, blood count analysis was performed. From these results, the SII, SIRI, PLR and NLR indices were determined. In case of symptom relapse after a successful course of biological treatment, patients were qualified for the treatment program once again and underwent the whole procedure for the second time. The data were collected based on the documentation gathered during each follow-up visit.

The urticaria activity score evaluated for 7 consecutive days (UAS7) is a commonly used diary-based measure that assesses the key sign (hives) and symptom (itch) of CSU. According to the EAACI/GA2LEN/EDF/WAO guidelines for urticaria UAS7 is recommended in clinical practice to determine disease activity and response to treatment. On the UAS scale, the patient assesses hive number and itch intensity once daily (every 24 h). A weekly score (UAS7) is calculated as the sum of the daily number of hives score and the itch severity score over 7 days. UAS7 values range from 0 to 42, with higher values indicating higher disease activity [11,12,13].

Dermatology life quality index (DLQI) is one of the most widely used tools to measure the impact of dermatological diseases on quality of life in adults and adolescents aged 16 and over. It consists of 10 questions concerning the quality of life of a patient with skin lesions one week prior to the survey. The respondent provides answers on a 4-point Likert scale ranging from 0 (not at all) to 3 (very strongly). The total score is obtained by adding the points for each question. Scores range from 0 to 30. The higher the score, the more impaired the patient quality of life is.

Data analysis was performed using statistical software JASP (version 0.12.1.). Calculated indicators and the results of standardized questionnaires were presented using basic descriptive statistics. The normality of distributions of the quantitative variables in the subgroups was confirmed using the Shapiro–Wilk test, on the basis of measures of skewness and kurtosis and by means of a visual assessment of histograms. A comparative analysis of the groups was performed using Student’s *t*-test (for quantitative variables with normal distribution) and the Mann–Whitney U test (for quantitative variables without normal distribution). The effectiveness of treatment was estimated using the of area under the graph of the curve obtained by connecting the points, which were the results of the UAS7 scale at successive measurements and calculated using trapezoidal rule. The lower the value, the higher the treatment efficacy (faster, stronger response to treatment). A paired sample *t*-test was introduced to compare the effectiveness of the treatment in subsequent treatment cycles. Spearman’s rho was used in correlation analysis. Mean differences (MD) between the groups were also determined at the 95% confidence interval (CI). A significance level of 0.05 was used in the calculations.

## 3. Results

Data from 56 patients of both sexes—41 (73.2%) female patients and 15 male patients aged 15 to 76 years (M = 41.48, SD = 16.85)—receiving OMA therapy for CSU were gathered. Of the study participants, 30 (53.57%) displayed a noticeable response to the implemented treatment (with UAS of 0 at the last visit) were classified as responders and 19 (33.93%), who had a weaker or no response (score greater than 0 on the UAS scale at the last visit), were classified as non-responders. Due to the early discontinuation of therapy, some of the data of 10 patients (18%) were missing, thus the majority of the final analysis results are based on 46 patients. The detailed descriptive statistics of the study group are presented in Table 1.

To assess whether the proposed indices, namely SII, SIRI, PLR and NLR, could be predictors of treatment efficacy (response to biological treatment), a comparison for independent samples was used. Due to deviations from the normality of the distribution of some variables, both the Student’s *t*-test and the Mann–Whitney test were used. Detailed results of the analyses are presented in Table 2 and Table 3.

Based on the above analysis, there were no statistically significant differences in the levels of the indicators observed. There was no statistically significant difference in the levels of SII, SIRI, PLR and NLR between responders and non-responders at the beginning or at the end of the treatment either. Although certain differences can be seen in the mean values of each index, the standard deviation and coefficient of variation for each of the index for both groups should be considered as high.

At the same time, a correlation analysis was performed and no significant correlation was found between UAS7 at the end of the treatment and the initial value of the proposed indices. However, a significant correlation was observed between urticaria severity (UAS7) and quality of life (DLQI). Results are presented in Table 4.

In further analyses, the quality of life was compared between the groups and at the beginning and the end of treatment in both groups. Significant differences were found in the level of quality of life between the groups at the end of the treatment. It is of note that both groups demonstrated statistically significant improvements in quality of life (even when OMA treatment was not fully effective). Detailed results are presented in Table 5 and Table 6.

In addition, the analysis of the treatment effectiveness in 24 patients (11 initial non-responders and 13 initial responders with symptoms relapse) undergoing OMA treatment was carried out again. Our analysis revealed significant differences in efficacy between the first and second treatment cycles, suggesting even higher efficacy in the second cycle (Table 7).

## 4. Discussion

The search for reliable predictors of treatment responses in CSU is one of the current hot topic research areas. The findings could provide additional information for the management of urticaria in clinical practice. The high effectiveness of OMA in patients’ refractory to antihistamines is already known [1]; however, the response is not always complete and sufficient. The systematic review, conducted by Fok et al., summarizes the role of IgE levels in the prediction of treatment response in CSU. The presented studies indicate that low levels of this immunoglobulin were associated with poor responses to OMA (strong evidence) but display good control under cyclosporine treatment [14].

There is an urgent need for further identification and confirmation of certain proposed biomarkers. According to the study conducted by Ertas et al., not only total IgE levels, but also their changes after 4 weeks of OMA treatment, have predictive value [15]. Other researchers examined the role of FcεRIreceptors. Their baseline levels were significantly lower in the group of patients with insufficient benefits from therapy [16]. In one of the more recent studies, authors reported the co-occurrence of anti-FcεRIIgE and IgG autoantibodies in cases of late or poor response to OMA treatment [17].

Some coagulation factors, such as D-dimer plasma values, are also being examined to determine the response to OMA treatment. Asero et al. noticed a dramatic decrease in D-dimer levels in the group of responders after the first administration of OMA. At the same time, no reduction in this parameter has been observed in non-responders [18]. In another paper, the same authors also demonstrated that the D-dimer plasma levels are much more frequently elevated in CSU patients, presenting an earlier response to OMA than in late responders and non-responders, suggesting that D-dimer plasma levels could be a positive predictive marker for anti-IgE therapy [19].

Researchers also examined different inflammatory markers in urticaria. The levels of CRP and IL-6 were elevated in CSU patients, in association with disease activity [7]. Furthermore, some researchers observed correlation between IL-6 levels and DLQI [20]. There were also studies concerning the role of IL-31. One showed an IL-31 decrease in patients who were successfully treated, whereas another study investigated the correlation between IL-31 secreting cells and the response to OMA [21,22]. Furthermore, researchers are also interested in the usefulness of simple, accessible blood tests, such as complete blood count [22]. Acer et al. showed the effect of OMA on hematologic and inflammatory parameters, such as increase in MPV and decrease in CRP, PLR and NLR levels [8]. Another study on OMA treatment also evaluated the above indexes along with MPV and their predictive role was put forward [5].

NLR and PLR were examined in various diseases and clinical states, also in the context of prognosis [23]. Two other inflammatory indexes—SIRI and SII—were indicated as biomarkers in different kinds of cancers or cardiovascular events [9,10]. SII was mentioned when evaluating prognosis of psoriasis [24]. Recent research has proposed the above parameters as new markers of response to OMA in CSU. The above indexes were described as accessible, cost-effective and predictive indicators [9]. As it has already been stated, further research is required to establish their true value in CSU.

In our study, we focused on low-cost and easy to obtain markers, which can be determined based on blood tests routinely collected throughout the course of treatment, according to the national health service guidelines for drug programs in Poland. Our analysis did not confirm SII, SIRI, PLR, or NLR as indicators/biomarkers of treatment response to OMA in CSU. However, the analysis of the mean values of the indexes may suggest the existence of some correlation between their levels and the prognosis of treatment efficacy. Unfortunately, considerable variation of the above indexes values in both responders and non-responders made it impossible to draw any definitive conclusions regarding their usefulness as biomarkers.

Furthermore, it should be pointed out that adopting the proposed indexes as biomarkers of treatment efficacy and patient enrollment based on them may lead to the exclusion of a significant number of patients who would ultimately respond to OMA treatment.

At the same time, it is worth noting significant improvement in the patients’ quality of life on OMA treatment. Patients experienced an improvement in quality of life despite not responding fully to treatment—even if the symptoms of the disease remained severe. It seems to be questionable to rely only on biological indicators as predictors of treatment response. Such an approach does not take into account a more holistic care of the patient, who—despite the lack of an objective cure—achieves a subjectively high improvement in the functioning and better everyday comfort of life. Such a situation could result from the treatment process itself, the care received, the interest shown by healthcare professionals or the hope of improving one’s condition.

In addition, it is noteworthy that patients from our group in subsequent treatment cycles responded even better than to the first OMA cycle. This indicates that CSU patients do not become resistant to OMA treatment. Furthermore, OMA therapy may even produce better results with subsequent treatment cycles. However, this relationship requires further research due to the small sample size in our study. In addition, it would be worthwhile to investigate how initial responders and non-responders react to subsequent treatment cycles.

It should also be highlighted that our study had some limitations. It is only one center study based on retrospective analysis. It was not possible to control the data collection process, which led to missing data from some patients, and as a result, a reduction in the size of the study group. At the same time, only data routinely collected as part of the procedures of the governmental treatment program in Poland were included in the analysis. When observing the results concerning the improvement in patients’ quality of life during treatment, it is worth considering extending the study to include other self-administered questionnaires or validated tools that can help identify factors influencing the aforementioned improvement. In addition, deviations from the normal distribution were found for certain variables, resulting in the need to apply non-parametric tests to the statistical analysis. More numerous patient groups could overcome this problem. We also observed a high rate of early treatment discontinuation, but due to the retrospective nature of the analysis it was not possible to determine the reasons for this issue. Treatment discontinuers included both patients who responded quickly to treatment (UAS7 score of 0 at the last recorded visit) and those for whom treatment was not effective. It is worth considering to collect information of treatment discontinuation causes in further studies.

## 5. Conclusions

A combination of one biomarker/biomarkers with clinical severity and maybe with psychological parameters of the patients in disease response to treatment could be of high value. Our results demonstrated the usefulness of quality of life assessment during OMA treatment. Quality of life evaluation provides additional information on benefits from therapy and a more holistic approach to the patient.

As it was mentioned, the response in the subsequent treatment cycles should be examined in further studies. Collecting additional samples from patients enrolled in the drug program to further expand the search for the effective and low-cost biomarkers of OMA efficacy is also worth considering.

In short:

1. Potential biomarkers should be evaluated on large groups of patients, preferably originating from more than one center.
2. A combination of biologic markers, clinical disease severity and psychological parameters should be considered in the efficacy of the treatment response to OMA in CSU patients.

## Figures and Tables

**Table 1:** Descriptive statistics.

Variable	N	Min	Max	Mean	SD
Age	56	15	76	41.48	16.85
UAS7					
1st visit	56	14	42	35.00	5.44
2nd visit	55	0	35	4.86	6.97
3rd visit	48	0	32	2.56	5.53
Dermatology Life Quality Index (DLQI)					
1st visit	56	9	30	19.21	5.06
2nd visit	55	0	14	2.33	3.74
3rd visit	48	0	11	1.00	2.09
Systemic Immune-inflammation Index (SII)					
1st visit	56	198.19	2771.58	752.74	475.94
2nd visit	55	156.19	1203.41	518.11	231.11
3rd visit	46	193.74	1208.89	560.91	258.07
Systemic Inflammation Response Index (SIRI)					
1st visit	56	0.30	8.06	1.36	1.21
2nd visit	55	0.27	2.31	0.99	0.53
3rd visit	46	0.39	3.66	1.106	0.61
Platelet/Lymphocyte Ratio (PLR)					
1st visit	56	53.62	264.23	158.53	52.71
2nd visit	55	70.98	250.89	141.96	43.92
3rd visit	46	77.40	321.21	144.50	46.66
Neutrophil/Lymphocyte Ratio (NLR)					
1st visit	56	0.79	9.27	2.57	1.54
2nd visit	55	0.76	4.61	1.91	0.80
3rd visit	46	0.81	3.90	2.00	0.74

**Table 2:** Comparison of index levels between groups (responders vs. non-responders).

Variable	Test	Statistic	*p*
SII start of treatment ^a^	Student	−0.04	0.305
	Mann–Whitney	228.00	0.249
SIRI start of treatment ^a^	Student	−0.34	0.739
	Mann–Whitney	261.50	0.637
PLR start of treatment	Student	−1.47	0.150
	Mann–Whitney	230.00	0.266
NLR start of treatment ^a^	Student	−0.22	0.830
	Mann–Whitney	273.00	0.813
SII end of treatment ^a^	Student	−0.04	0.968
	Mann–Whitney	258.00	0.903
SIRI end of treatment ^a^	Student	−1.21	0.234
	Mann–Whitney	207.50	0.322
PLR end of treatment ^a^	Student	−0.23	0.820
	Mann–Whitney	235.00	0.713
NLR end of treatment ^a^	Student	0.56	0.581
	Mann–Whitney	244.50	0.875

^a^ Significant deviations from normality of distribution were found. It is recommended to take the results of the Mann–Whitney test.

**Table 3:** Indicator values by group (responders vs. non-responders).

	N	Mean	SD	SE
SII start of treatment				
Non-responders	19	670.87	421.88	96.79
Responders	30	823.16	542.74	99.27
SIRI start of treatment				
Non-responders	19	1.29	1.01	0.23
Responders	30	1.41	1.43	0.26
PLR start of treatment				
Non-responders	19	146.95	49.4	11.33
Responders	30	169.69	55.06	10.05
NLR start of treatment				
Non-responders	19	2.53	1.59	0.37
Responders	30	2.63	1.68	0.31
SII end of treatment				
Non-responders	18	558.97	245.09	57.78
Responders	28	562.16	270.49	51.12
SIRI end of treatment				
Non-responders	18	0.97	0.44	0.10
Responders	28	1.19	0.69	0.13
PLR end of treatment				
Non-responders	18	142.52	41.70	9.83
Responders	28	145.78	50.29	9.51
NLR end of treatment				
Non-responders	18	2.08	0.90	0.21
Responders	28	1.96	0.63	0.12

**Table 4:** Correlation of selected variables.

Variable		UAS7 End of Treatment
SII start of treatment	Spearman’s rho	−0.203
	*p*	0.166
SIRI start of treatment	Spearman’s rho	−0.128
	*p*	0.385
PLR start of treatment	Spearman’s rho	−0.162
	*p*	0.272
NLR start of treatment	Spearman’s rho	−0.059
	*p*	0.693
DLQI end of treatment	Spearman’s rho	0.785
	*p*	<0.001

**Table 5:** Comparison of quality of life levels between groups.

Variable	Test	Statistic	*p*
DLQI start of treatment	Student	−0.39	0.699
	Mann–Whitney	269.50	0.757
DLQI end of treatment ^a^	Student	4.29	<0.001
	Mann–Whitney	478.00	<0.001

^a^ Significant deviations from normality of distribution were found; it is recommended to take the results of the Mann–Whitney test.

**Table 6:** Comparison of the changes in quality of life before and after the treatment in both groups.

Group	T	*p*	DLQI Start	SD	DLQI End	SD
Non-Responders	15.27	<0.001	19.32	4.39	2.37	2.83
Responders	19.81	<0.001	19.90	5.52	0.1	0.31

**Table 7:** Comparison of efficacy in treatment cycles.

Variable	N	Mean	SD	SE	t	df	*p*
1st cycle efficacy—2nd cycle efficacy					2.153	24	0.042
1st cycle efficacy	24	53.15	28.26	5.77			
2nd cycle efficacy	24	41.79	28.55	5.83			

## Data Availability

The dataset for this research can be found at shorturl.at/dqrI1.
